# Crystal structure of ethyl (6-hy­droxy-1-benzo­furan-3-yl)acetate sesquihydrate

**DOI:** 10.1107/S1600536814024349

**Published:** 2014-11-21

**Authors:** G. Krishnaswamy, P. A. Suchetan, S. Sreenivasa, S. Naveen, N. K. Lokanath, D. B. Aruna Kumar

**Affiliations:** a Department of Studies and Research in Chemistry, Tumkur University, Tumkur 572 103, India; b Institution of Excellence, Vijnana Bhavan, University of Mysore, Manasagangotri, Mysore 570 006, India; c Department of Studies in Physics, University of Mysore, Manasagangotri, Mysore 570 006, India

**Keywords:** crystal structure, hydrates, O—H⋯O hydrogen bonds, C—H⋯π inter­actions, benzo­furan

## Abstract

In the title hydrate, C_12_H_12_O_4_·1.5H_2_O, one of the water mol­ecules in the asymmetric unit is located on a twofold rotation axis. The mol­ecule of the benzo­furan derivative is essentially planar (r.m.s. deviation for the non-H atoms = 0.021 Å), with the ester group adopting a fully extended conformation. In the crystal, O—H⋯O hydrogen bonds between the water mol­ecules and the hy­droxy groups generate a centrosymmetric *R*_6_^6^(12) ring motif. These *R*_6_^6^(12) rings are fused, forming a one-dimensional motif extending along the *c*-axis direction.

## Abstract 2

The crystal structure of ethyl (6-hy­droxy-1-benzo­furan-3-yl)acetate sesquihydrate exhibits a one-dimensional hydrogen-bond motif consisting of 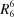
(12) rings joined at water mol­ecules located on a twofold rotation axis.

## Chemical context   

Furan heterocycles are of inter­est for synthetic chemists as they possess various pharmacological and biological activities including anti­tuberculosis (Tawari *et al.*, 2010 ▶), anti-inflammatory (Shin *et al.*, 2011 ▶) and anti­bacterial (Kirilmis *et al.*, 2008 ▶) activity. Substituted benzo­furans have found applications as fluorescent sensors (Oter *et al.*, 2007 ▶), anti-oxidants, brightening agents and drugs. Moreover, benzo­furan carb­oxy­lic acid ethyl ester also exhibits selective cytotoxicity against a tumorigenic cell line (Hayakawa *et al.*, 2004 ▶). In view of the above facts, and as a continuation of our structural studies on benzo­furans (Arunakumar, Krishnaswamy *et al.*, 2014 ▶; Arunakumar, Desai Nivedita *et al.*, 2014 ▶), the title compound has been synthesized, characterized by FT IR, ^1^H NMR and LC–MS methods and its crystal structure determined.
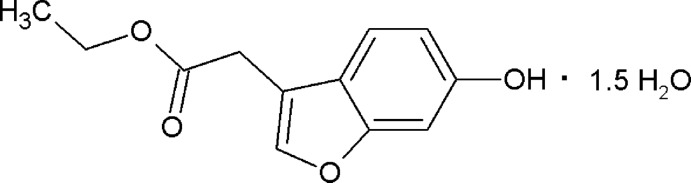


## Structural commentary   

**Figure 1:**
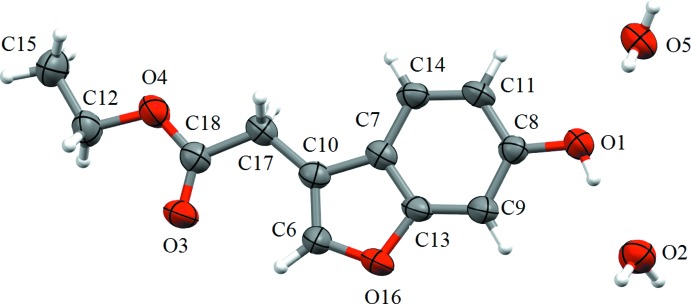
The mol­ecular structure of the title compound. Displacement ellipsoids are drawn at the 50% probability level.

The title compound crystallizes as a 1.5-hydrate with one of the symmetry-independent water mol­ecules occupying a special position of *C*_2_ symmetry. The mol­ecular structure of the title compound is shown in Fig. 1 ▶. The mol­ecule is almost planar (r.m.s. deviation for the non-H atoms = 0.021 Å) and the ethyl acetate fragment adopts a fully extended conformation.

## Supra­molecular features   

**Figure 2:**
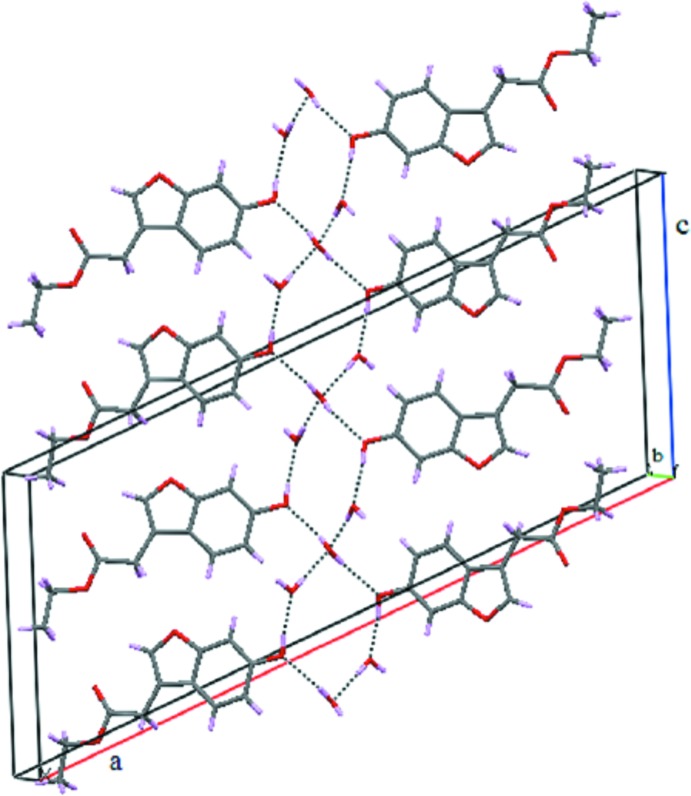
Hydrogen-bonding inter­actions (dashed lines) featuring a fused 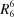
(12) ring motif.

**Figure 3:**
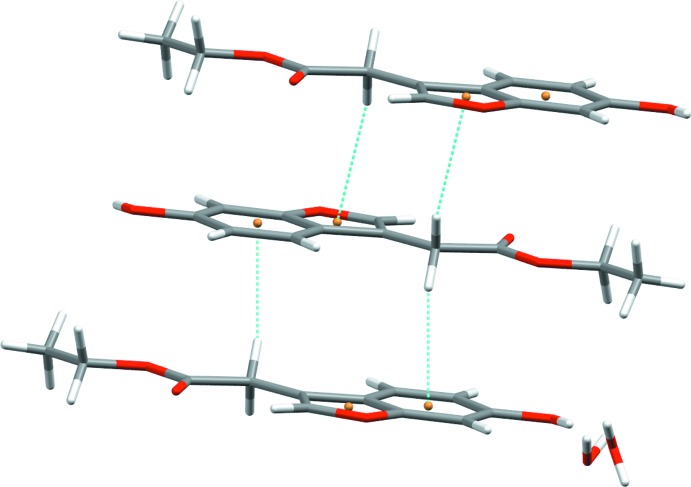
The C—H⋯π inter­actions (dashed lines) in the title compound.

Hydrogen bonds (Table 1 ▶) between two hy­droxy groups and four water mol­ecules generate a centrosymmetric 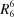
(12) ring motif. The rings are fused at the position of the O5 atoms, *i.e.* through water mol­ecules located at special positions. In effect, two anti­parallel chains of hydrogen bonds are formed that are fused at every fourth O atom and which propagate along the crystallographic *c-*axis (Fig. 2 ▶). In the crystal, the components are connected into a three-dimensional network through additional hydrogen bonds between the water mol­ecule in a general position and the ester carbonyl group. In addition to strong hydrogen bonds, weaker C—H⋯π inter­actions are observed between the methyl­ene group H atoms and the benzene and furan rings (Fig. 3 ▶ and Table 1 ▶).

## Synthesis and crystallization   

2-(6-Hy­droxy-1-benzo­furan-3-yl)acetic acid (2.0 g, 0.010 mmol) was taken in a round-bottomed flask containing ethanol (10 mL). Concentrated sulfuric acid (1 mL) was added and the reaction mixture was refluxed for 4 h at 353 K. After completion of the reaction, the reaction mixture was poured into ice-cold water and extracted to an ethyl acetate layer. The organic layer was washed with water followed by brine solution and dried over anhydrous sodium sulfate. The organic layer was concentrated under vacuum, giving a reddish residue. The residue was purified by column chromatography using silica gel (60–120 mesh) and ethyl acetate/petroleum ether (2:8) as eluent, affording a colourless crystalline product. Crystals suitable for X-ray analysis were formed by slow evaporation of the solution of the compound in ethyl acetate and petroleum ether (3:2) at room temperature. As the product had been water worked-up, water might have entered in the solid interstices during work-up.

## Refinement   

Crystal data, data collection and structure refinement details are summarized in Table 2 ▶. The H atoms of water mol­ecules were located from a difference Fourier map. The H atom bound to O5 was freely refined and those bound to O2 had the O—H distances restrained to 0.85 (2) Å. The remaining C/O-bound H atoms were fixed geometrically (C—H = 0.93–0.97 and O—H = 0.82 Å) and allowed to ride on their parent atoms with *U*_iso_(H) = 1.5*U*_eq_(C,O) for methyl and hy­droxy H atoms, and 1.2*U*_eq_(C) for other H atoms.

## Supplementary Material

Crystal structure: contains datablock(s) I. DOI: 10.1107/S1600536814024349/gk2620sup1.cif

Structure factors: contains datablock(s) I. DOI: 10.1107/S1600536814024349/gk2620Isup2.hkl

Supporting information file. DOI: 10.1107/S1600536814024349/gk2620Isup3.cml

CCDC reference: 1032887

Additional supporting information:  crystallographic information; 3D view; checkCIF report

## Figures and Tables

**Table 1:** Hydrogen-bond geometry (, ) *Cg*1 is the centroid of the C7/C13/C9/C8/C11/C14 benzene ring and *Cg*2 is the centroid of the O16/C6/C10/C7/C13 furan ring.

*D*H*A*	*D*H	H*A*	*D* *A*	*D*H*A*
O1H1O2	0.82	1.88	2.692(4)	170
O2H2*A*O3^i^	0.85	1.96	2.788(4)	164
O2H2*B*O5^ii^	0.85	2.00	2.844(4)	174
O5H5O1^iii^	0.85(4)	2.09(4)	2.870(3)	152(4)
C17H17*B* *Cg*1^iv^	0.97	2.68	3.485(3)	140
C17H17*A* *Cg*2^v^	0.97	2.99	3.889(3)	154

Symmetry codes: (i) 
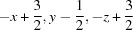
; (ii) 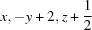; (iii) 
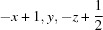
; (iv) 
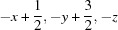
; (v) 
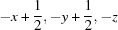
.

**Table 2:** Experimental details

Crystal data	
Chemical formula	2C_12_H_12_O_4_3H_2_O
*M*_r_	494.48
Crystal system, space group	Monoclinic, *C*2/*c*
Temperature (K)	296
*a*, *b*, *c* ()	29.191(6), 7.3291(17), 12.587(3)
()	113.074(13)
*V* (^3^)	2477.4(9)
*Z*	4
Radiation type	Cu *K*
(mm^1^)	0.89
Crystal size (mm)	0.47 0.34 0.26

Data collection	
Diffractometer	Bruker APEXII
Absorption correction	Multi-scan (*SADABS*; Bruker, 2009 ▶)
*T*_min_, *T*_max_	0.730, 0.793
No. of measured, independent and observed [*I* > 2(*I*)] reflections	8441, 1968, 1132
*R*_int_	0.114
(sin /)_max_ (^1^)	0.584

Refinement	
*R*[*F* ^2^ > 2(*F* ^2^)], *wR*(*F* ^2^), *S*	0.067, 0.224, 1.06
No. of reflections	1968
No. of parameters	168
No. of restraints	1
H-atom treatment	H atoms treated by a mixture of independent and constrained refinement
_max_, _min_ (e ^3^)	0.40, 0.54

Computer programs: *APEX2*, *SAINT-Plus* and *XPREP* (Bruker, 2009 ▶), *SHELXS97* and *SHELXL97* (Sheldrick, 2008 ▶) and *Mercury* (Macrae *et al.*, 2008 ▶).

## supplementary crystallographic information

### Crystal data

**Table:**

2C_12_H_12_O_4_·3H_2_O	prism
*M**_r_* = 494.48	*D*_x_ = 1.326 Mg m^−^^3^
Monoclinic, *C*2/*c*	Melting point: 447 K
Hall symbol: -C 2yc	Cu *K*α radiation, λ = 1.54178 Å
*a* = 29.191 (6) Å	Cell parameters from 125 reflections
*b* = 7.3291 (17) Å	θ = 6.3–64.2°
*c* = 12.587 (3) Å	µ = 0.89 mm^−^^1^
β = 113.074 (13)°	*T* = 296 K
*V* = 2477.4 (9) Å^3^	Prism, colourless
*Z* = 4	0.47 × 0.34 × 0.26 mm
*F*(000) = 1048	

### Data collection

**Table:**

Bruker APEXII diffractometer	1968 independent reflections
Radiation source: fine-focus sealed tube	1132 reflections with *I* > 2σ(*I*)
Graphite monochromator	*R*_int_ = 0.114
phi and φ scans	θ_max_ = 64.2°, θ_min_ = 6.3°
Absorption correction: multi-scan (*SADABS*; Bruker, 2009)	*h* = −31→33
*T*_min_ = 0.730, *T*_max_ = 0.793	*k* = −8→8
8441 measured reflections	*l* = −13→13

### Refinement

**Table:**

Refinement on *F*^2^	Primary atom site location: structure-invariant direct methods
Least-squares matrix: full	Secondary atom site location: difference Fourier map
*R*[*F*^2^ > 2σ(*F*^2^)] = 0.067	Hydrogen site location: inferred from neighbouring sites
*wR*(*F*^2^) = 0.224	H atoms treated by a mixture of independent and constrained refinement
*S* = 1.06	*w* = 1/[σ^2^(*F*_o_^2^) + (0.1328*P*)^2^] where *P* = (*F*_o_^2^ + 2*F*_c_^2^)/3
1968 reflections	(Δ/σ)_max_ < 0.001
168 parameters	Δρ_max_ = 0.40 e Å^−^^3^
1 restraint	Δρ_min_ = −0.54 e Å^−^^3^

### Special details

**Table:**

Experimental. Thin-layer chromatography (TLC) was carried out on Merck pre-coated silica gel plates to monitor the progress of the reaction. The FT–IR spectra were recorded as KBr pellets using JASCO FT–IR-4100 spectrophotometer in the range 4000–400 cm^-1^ at a resolution of 2 cm^-1^. ^1^H NMR spectra were recorded in CDCl_3_ and DMSO-d_6_ on a JEOL-400 MHz NMR instrument. Chemical shifts are reported in δ values in parts per million relative to TMS. Mass spectral data were obtained on an Agilent LC–MS column C-18 instrument.The IR spectrum of (I) exhibits strong bands at 1686 cm^-1^ and 1193 cm^-1^ due to C=O and C-O stretchings, respectively. A single band appearing at 3340 cm^-1^ is due to OH group stretching. Appearance of bands in the range 3011–2907 cm^-1^ is due to aromatic stretching and bands in the range 2970–2815 cm^-1^ are due to C—H stretching, thus confirming the presence of the saturated hydrocarbons in (I).The ^1^H NMR spectrum of (I) shows peaks at δ 9.53 (s, 1H, Ar-OH), 6.69 (s, 1H, furan-H), 7.35–7.33 (d, 1H, Ar-H), 6.88–6.87 (d, 1H, Ar-H), 6.75–6.72 (q, 1H, Ar-H), 4.12–4.07 (q, 2H, OCH_2_), 3.34 (s, 2H, CH_2_), 1.12–1.17 (t, 3H, CH_3_). The LC–MS spectrum shows the appearance of molecular ion peaks at m/z 221 and 222 values, confirming the structure of the compound.
Geometry. All s.u.'s (except the s.u. in the dihedral angle between two l.s. planes) are estimated using the full covariance matrix. The cell s.u.'s are taken into account individually in the estimation of s.u.'s in distances, angles and torsion angles; correlations between s.u.'s in cell parameters are only used when they are defined by crystal symmetry. An approximate (isotropic) treatment of cell s.u.'s is used for estimating s.u.'s involving l.s. planes.
Refinement. Refinement of *F*^2^ against ALL reflections. The weighted *R*-factor *wR* and goodness of fit *S* are based on *F*^2^, conventional *R*-factors *R* are based on *F*, with *F* set to zero for negative *F*^2^. The threshold expression of *F*^2^ > 2σ(*F*^2^) is used only for calculating *R*-factors(gt) *etc*. and is not relevant to the choice of reflections for refinement. *R*-factors based on *F*^2^ are statistically about twice as large as those based on *F*, and *R*- factors based on ALL data will be even larger.

### Fractional atomic coordinates and isotropic or equivalent isotropic displacement parameters (Å^2^)

**Table:**

	*x*	*y*	*z*	*U*_iso_*/*U*_eq_	
O16	0.74036 (8)	0.9708 (4)	0.7193 (2)	0.0605 (10)	
C7	0.71590 (10)	0.9610 (4)	0.5264 (3)	0.0378 (10)	
C8	0.61898 (11)	0.8688 (4)	0.4921 (3)	0.0391 (11)	
C9	0.65308 (11)	0.8957 (4)	0.6055 (3)	0.0448 (11)	
H9	0.6444	0.8846	0.6689	0.054*	
C10	0.76857 (11)	1.0071 (4)	0.5752 (3)	0.0389 (10)	
C11	0.63322 (12)	0.8851 (4)	0.3998 (3)	0.0458 (11)	
H11	0.6097	0.8646	0.3255	0.055*	
C12	0.92468 (12)	1.1583 (5)	0.5340 (3)	0.0523 (12)	
H12A	0.9449	1.0616	0.5824	0.063*	
H12B	0.9307	1.2693	0.5793	0.063*	
C13	0.70057 (11)	0.9401 (4)	0.6160 (3)	0.0405 (11)	
C14	0.68085 (12)	0.9305 (4)	0.4142 (3)	0.0427 (11)	
H14	0.6897	0.9409	0.3510	0.051*	
C15	0.93746 (14)	1.1861 (6)	0.4312 (4)	0.0680 (15)	
H15A	0.9289	1.0786	0.3839	0.102*	
H15B	0.9725	1.2092	0.4564	0.102*	
H15C	0.9191	1.2884	0.3873	0.102*	
C17	0.79811 (11)	1.0380 (4)	0.5048 (3)	0.0420 (11)	
H17A	0.7819	1.1334	0.4495	0.050*	
H17B	0.7967	0.9275	0.4612	0.050*	
C18	0.85173 (12)	1.0893 (4)	0.5659 (3)	0.0410 (11)	
O1	0.57017 (8)	0.8246 (4)	0.4691 (2)	0.0512 (9)	
H1	0.5667	0.8083	0.5301	0.077*	
O2	0.54997 (10)	0.7984 (4)	0.6597 (2)	0.0639 (10)	
H2A	0.5687	0.7310	0.7144	0.096*	
H2B	0.5365	0.8790	0.6866	0.096*	
O3	0.87331 (9)	1.1106 (3)	0.6691 (2)	0.0586 (9)	
O4	0.87249 (9)	1.1095 (3)	0.4913 (2)	0.0523 (9)	
O5	0.5000	0.9512 (5)	0.2500	0.0598 (13)	
C6	0.77984 (12)	1.0112 (5)	0.6912 (3)	0.0526 (12)	
H6	0.8114	1.0388	0.7458	0.063*	
H5	0.4849 (19)	0.879 (5)	0.194 (3)	0.104 (18)*	

### Atomic displacement parameters (Å^2^)

**Table:**

	*U*^11^	*U*^22^	*U*^33^	*U*^12^	*U*^13^	*U*^23^
O16	0.0473 (13)	0.0952 (19)	0.0313 (16)	−0.0166 (11)	0.0069 (14)	−0.0131 (13)
C7	0.0405 (15)	0.0324 (14)	0.036 (2)	0.0010 (10)	0.0098 (17)	0.0003 (14)
C8	0.0393 (15)	0.0418 (16)	0.035 (2)	0.0035 (10)	0.0137 (17)	0.0009 (15)
C9	0.0420 (16)	0.0557 (18)	0.034 (2)	0.0000 (12)	0.0121 (17)	−0.0065 (16)
C10	0.0428 (15)	0.0362 (15)	0.036 (2)	−0.0021 (10)	0.0130 (17)	−0.0033 (15)
C11	0.0424 (16)	0.0578 (19)	0.028 (2)	0.0010 (12)	0.0038 (18)	0.0009 (16)
C12	0.0406 (17)	0.060 (2)	0.054 (3)	−0.0104 (12)	0.0155 (19)	0.0029 (19)
C13	0.0419 (16)	0.0455 (16)	0.029 (2)	−0.0013 (12)	0.0082 (17)	−0.0069 (15)
C14	0.0474 (16)	0.0490 (17)	0.029 (2)	0.0053 (12)	0.0125 (17)	0.0032 (15)
C15	0.059 (2)	0.088 (3)	0.064 (3)	−0.0184 (18)	0.032 (2)	−0.005 (2)
C17	0.0401 (15)	0.0410 (16)	0.038 (2)	−0.0023 (11)	0.0077 (16)	−0.0006 (15)
C18	0.0442 (16)	0.0346 (15)	0.042 (2)	−0.0033 (11)	0.0148 (19)	0.0008 (15)
O1	0.0382 (11)	0.0702 (15)	0.0411 (15)	−0.0032 (9)	0.0112 (12)	0.0030 (14)
O2	0.0591 (15)	0.0818 (19)	0.0477 (17)	0.0184 (11)	0.0176 (14)	0.0097 (15)
O3	0.0555 (14)	0.0800 (17)	0.0344 (16)	−0.0188 (11)	0.0114 (14)	−0.0080 (13)
O4	0.0423 (12)	0.0624 (14)	0.0454 (16)	−0.0119 (9)	0.0100 (13)	0.0003 (12)
O5	0.0500 (18)	0.065 (2)	0.053 (3)	0.000	0.008 (2)	0.000
C6	0.0398 (17)	0.079 (2)	0.040 (2)	−0.0127 (14)	0.0166 (18)	−0.017 (2)

### Geometric parameters (Å, º)

**Table:**

O16—C6	1.364 (5)	C12—H12A	0.9700
O16—C13	1.381 (3)	C12—H12B	0.9700
C7—C13	1.375 (5)	C14—H14	0.9300
C7—C14	1.399 (4)	C15—H15A	0.9600
C7—C10	1.454 (4)	C15—H15B	0.9600
C8—O1	1.377 (4)	C15—H15C	0.9600
C8—C11	1.385 (6)	C17—C18	1.496 (4)
C8—C9	1.397 (4)	C17—H17A	0.9700
C9—C13	1.379 (5)	C17—H17B	0.9700
C9—H9	0.9300	C18—O3	1.211 (4)
C10—C6	1.365 (6)	C18—O4	1.309 (5)
C10—C17	1.476 (5)	O1—H1	0.8200
C11—C14	1.371 (6)	O2—H2A	0.8499
C11—H11	0.9300	O2—H2B	0.8500
C12—O4	1.448 (4)	O5—H5	0.854 (19)
C12—C15	1.494 (6)	C6—H6	0.9300
			
C6—O16—C13	106.0 (3)	C11—C14—C7	118.4 (4)
C13—C7—C14	117.7 (3)	C11—C14—H14	120.8
C13—C7—C10	108.0 (3)	C7—C14—H14	120.8
C14—C7—C10	134.2 (4)	C12—C15—H15A	109.5
O1—C8—C11	118.1 (3)	C12—C15—H15B	109.5
O1—C8—C9	120.8 (4)	H15A—C15—H15B	109.5
C11—C8—C9	121.1 (3)	C12—C15—H15C	109.5
C13—C9—C8	114.7 (4)	H15A—C15—H15C	109.5
C13—C9—H9	122.6	H15B—C15—H15C	109.5
C8—C9—H9	122.6	C10—C17—C18	118.0 (3)
C6—C10—C7	103.2 (4)	C10—C17—H17A	107.8
C6—C10—C17	133.3 (3)	C18—C17—H17A	107.8
C7—C10—C17	123.5 (3)	C10—C17—H17B	107.8
C14—C11—C8	122.2 (3)	C18—C17—H17B	107.8
C14—C11—H11	118.9	H17A—C17—H17B	107.1
C8—C11—H11	118.9	O3—C18—O4	124.2 (3)
O4—C12—C15	107.3 (3)	O3—C18—C17	125.6 (4)
O4—C12—H12A	110.3	O4—C18—C17	110.2 (3)
C15—C12—H12A	110.3	C8—O1—H1	109.5
O4—C12—H12B	110.3	H2A—O2—H2B	109.5
C15—C12—H12B	110.3	C18—O4—C12	118.5 (3)
H12A—C12—H12B	108.5	O16—C6—C10	113.5 (3)
C7—C13—C9	125.9 (3)	O16—C6—H6	123.3
C7—C13—O16	109.3 (3)	C10—C6—H6	123.3
C9—C13—O16	124.9 (4)		
			
O1—C8—C9—C13	−179.7 (3)	C6—O16—C13—C9	−179.7 (3)
C11—C8—C9—C13	0.5 (4)	C8—C11—C14—C7	0.0 (4)
C13—C7—C10—C6	−0.6 (3)	C13—C7—C14—C11	1.2 (4)
C14—C7—C10—C6	−178.3 (3)	C10—C7—C14—C11	178.8 (3)
C13—C7—C10—C17	179.3 (3)	C6—C10—C17—C18	−1.7 (5)
C14—C7—C10—C17	1.6 (5)	C7—C10—C17—C18	178.5 (3)
O1—C8—C11—C14	179.3 (3)	C10—C17—C18—O3	−1.1 (5)
C9—C8—C11—C14	−0.9 (5)	C10—C17—C18—O4	179.3 (2)
C14—C7—C13—C9	−1.7 (5)	O3—C18—O4—C12	0.7 (5)
C10—C7—C13—C9	−179.9 (3)	C17—C18—O4—C12	−179.6 (2)
C14—C7—C13—O16	178.4 (2)	C15—C12—O4—C18	−175.7 (3)
C10—C7—C13—O16	0.2 (3)	C13—O16—C6—C10	−0.6 (4)
C8—C9—C13—C7	0.8 (4)	C7—C10—C6—O16	0.7 (4)
C8—C9—C13—O16	−179.3 (3)	C17—C10—C6—O16	−179.2 (3)
C6—O16—C13—C7	0.2 (3)		

### Hydrogen-bond geometry (Å, º)

Cg1 is the centroid of the C7/C13/C9/C8/C11/C14 benzene ring and Cg2 is the centroid of the O16/C6/C10/C7/C13 furan ring.

**Table:**

*D*—H···*A*	*D*—H	H···*A*	*D*···*A*	*D*—H···*A*
O1—H1···O2	0.82	1.88	2.692 (4)	170
O2—H2*A*···O3^i^	0.85	1.96	2.788 (4)	164
O2—H2*B*···O5^ii^	0.85	2.00	2.844 (4)	174
O5—H5···O1^iii^	0.85 (4)	2.09 (4)	2.870 (3)	152 (4)
C17—H17*B*···*Cg*1^iv^	0.97	2.68	3.485 (3)	140
C17—H17*A*···*Cg*2^v^	0.97	2.99	3.889 (3)	154

Symmetry codes: (i) −*x*+3/2, *y*−1/2, −*z*+3/2; (ii) *x*, −*y*+2, *z*+1/2; (iii) −*x*+1, *y*, −*z*+1/2; (iv) −*x*+1/2, −*y*+3/2, −*z*; (v) −*x*+1/2, −*y*+1/2, −*z*.
